# Structural Design and Electromagnetic Performance Analysis of Octupole Active Radial Magnetic Bearing

**DOI:** 10.3390/s24248200

**Published:** 2024-12-22

**Authors:** Qixuan Zhu, Yujun Lu, Zhongkui Shao

**Affiliations:** 1 School of Mechanical Engineering, Zhejiang Sci-Tech University, Hangzhou 310018, China; 202230503330@mails.zstu.edu.cn; 2 Longgang Institute of Zhejiang Sci-Tech University, Wenzhou 325802, China; 3 Zhejiang Institute of Mechanical & Electrical Engineering Co., Ltd., Hangzhou 310051, China; shaozk@zjimee.com.cn

**Keywords:** magnetic bearing, structural design, electromagnetic performance, Ansys Maxwell

## Abstract

This study addresses the challenges of magnetic circuit coupling and control complexity in active radial magnetic bearings (ARMBs) by systematically investigating the electromagnetic performance of four magnetic pole configurations (NNSS, NSNS, NNNN, and SSSS). Initially, equivalent magnetic circuit modeling and finite element analysis (FEA) were employed to analyze the magnetic circuit coupling phenomena and their effects on the magnetic flux density distribution for each configuration. Subsequently, the air gap flux density and electromagnetic force were quantified under rotor eccentricity caused by unbalanced disturbances, and the dynamic performances of the ARMBs were evaluated for eccentricity along the x-axis and at 45°. Finally, experiments measured the electromagnetic forces acting on the rotor under the NNSS and NSNS configurations during eccentric conditions. The results indicate that the NNSS configuration significantly reduces magnetic circuit coupling, improves the uniformity of electromagnetic force distribution, and offers superior stability and control efficiency under asymmetric conditions. Experimental results deviated by less than 10% from the simulations, confirming the reliability and practicality of the proposed design. These findings provide valuable insights for optimizing ARMB pole configurations and promote their application in high-speed, high-precision industrial fields such as aerospace and power engineering.

## 1. Introduction

Magnetic bearings are a high-performance support technology. They have tremendous potential for use in high-speed rotating machinery because of their lack of contact and friction, excellent accuracy, absence of lubrication, and extended service life [1,2]. With the advancement of industrial technology, conventional mechanical contact bearings have failed to fulfill the increased speed and power density demands of motors in current drive systems [3,4,5,6]. In this setting, high-performance magnetic bearings have emerged as a fundamental technology for boosting speed and power output [7,8,9,10,11,12], notably in power generation, aerospace, manufacturing, and other critical research and application industries [13,14,15,16,17,18].

Magnetic bearings can be divided into three types: hybrid, passive, and active [19,20]. The direction of the force on the bearing determines whether the magnetic bearing is radial or axial. Active radial magnetic bearings (ARMBs) are highly versatile and find extensive use in the ultra-high-speed motor industry because of their compact size, minimal power consumption, and exceptional levitation force density [21,22,23,24,25]. ARMBs generate a control current by regulating the current in an electromagnetic coil. If the rotor is not centered, the electromagnetic force from the stator coil can be used to recenter the rotor and establish dynamically stable levitation [26,27].

However, ARMB technology development is facing severe magnetic couplings, complicated control systems, and high-performance parameters. The distribution pattern of the magnetic pole is the key to the design of the bearing structure. It has a direct influence on the electromagnetic field distribution, the density of the magnetic flux, the electromagnetic force, the magnetic coupling phenomena between pairs of poles, and the complex control methods [28,29,30]. Generally, ARMBs adopt an octupole distribution. Through the control current in different directions between the magnetic poles, four kinds of magnetic pole configurations can be developed: NNSS, NSNS, NNNN, and SSSS. These magnetic pole configurations have a significant impact on bearing performance, such as the magnitude of the electromagnetic force and flux density at the balance and offset of the rotor, the distribution of magnetic lines, and the magnetic coupling phenomenon between magnetic poles.

In the 1990s, NNSS pole configurations were widely investigated in the field of magnetic levitation bearings [31], which are notable for their reduced flux leakage and increased load capacity. However, the conventional design still has obvious deficiencies in terms of complex control requirements and magnetic coupling phenomena, especially in the case of unbalanced rotor disturbances. To address these issues, this paper combines FEM simulation and experimental verification methods to quantify the air gap flux density and electromagnetic force performance when the rotor is deflected by an unbalanced disturbance, and this study provides a reference design for improving the stability and control efficiency of high-performance rotating equipment. Li et al. [32] simulated and optimized the magnetic field uniformity and electromagnetic force output of the bearing by changing the number of magnetic poles, but did not systematically analyze the magnetic circuit coupling phenomenon under different magnetic pole configurations. In this work, a comprehensive comparative analysis of magnetic circuit coupling in four pole configurations was conducted, providing valuable insights for selecting optimal configurations in magnetic bearing designs. Additionally, the electromagnetic performance of the rotor under various eccentricity levels along the x-axis and at a 45° offset was evaluated, revealing the superior performance of the NNSS configuration under asymmetric eccentric conditions. These findings offer a novel perspective for the application of magnetic bearings in complex operating environments. Kandil et al. [33] investigated the impact of pole configuration angles on the control process of oscillatory rotors in ARMBs. However, their study focused primarily on open-loop control systems, without fully analyzing the effects of pole configurations on system complexity and control precision in closed-loop systems. Furthermore, their experiments and simulations were conducted under ideal conditions, leaving the performance of ARMBs under real-world scenarios, such as rotor eccentricity and imbalance, unexplored. This study addresses these limitations by employing theoretical modeling and FEA to demonstrate the advantages of the NNSS pole configuration in closed-loop control systems. The NNSS configuration not only improves system stability but also reduces control complexity, making it particularly suitable for high-speed and unbalanced operating conditions. Experimental validation under actual eccentric conditions confirmed the NNSS configuration’s superior stability and control efficiency, providing practical guidance for the design of high-performance magnetic bearings. Ye et al. [34] designed a novel four-degree-of-freedom homopolar hybrid magnetic levitation bearing using a combination of permanent magnets and electromagnets to improve the static and dynamic performance of the magnetic levitation system. Although homopolar design improves the magnetic field decoupling between the degrees of freedom, the magnetic coupling phenomenon may still exist for complex working conditions. The study focuses on the optimization of the homopolar structure and does not provide a systematic analysis of the performance differences between the different magnetic pole fractions. Due to the hybrid design combining permanent magnets and electromagnets, the system may require more complex control strategies, which increases the complexity of hardware and algorithms, and the study does not sufficiently consider the impact of actual working conditions such as rotor eccentricity or imbalance on the system performance. Our study focuses on an all-electromagnetic design that utilizes the NNSS pole configuration to significantly reduce the magnetic coupling phenomenon, especially in the case where the rotor undergoes eccentricity conditions. Our NNSS configuration avoids the introduction of permanent magnets, which significantly reduces the complexity of the control strategy and hardware design, and the performance of the NNSS configuration under eccentric and unbalanced conditions is systematically analyzed through simulations and experiments, demonstrating that it has superior dynamic stability and control efficiency. Noh et al. [35] designed a radial magnetic bearing with nine-pole asymmetric pole distribution with the aim of improving the efficiency of electromagnetic force utilization. Although the nine-pole design reduces the magnetic circuit coupling to a certain extent, the increase in the number of magnetic poles may lead to a rise in the complexity of system design, fabrication, and control, and their study mainly focuses on the performance under ideal operating conditions and lacks in-depth analyses of rotor eccentricity or dynamic unbalance cases. The experimental validation mainly focuses on the unbiased control strategy and does not comprehensively analyze the effect of the magnetic pole configuration on the distribution of electromagnetic force in the magnetic bearings and the key performance of control complexity. Our study systematically analyses the electromagnetic force distribution and control performance of the NNSS configuration under different eccentricity directions and proves that it has higher control accuracy and stability under eccentric and unbalanced conditions. Through extensive simulation and experimental verification, we demonstrate that the NNSS configuration not only maintains high control accuracy, but also significantly reduces the complexity of design and operation, making it more suitable for high-speed and dynamic industrial applications.

The purpose of this paper is not only to design an ARMB with NNSS pole configuration, but also to provide an in-depth analysis of the electromagnetic performance of both NNSS and NSNS pole configurations under different eccentricities and dynamic operating conditions. Simulation and experimental results show that the application of NNSS configuration significantly reduces the magnetic circuit coupling phenomenon, improves the stability of the system, and reduces the complexity of system design and operation. The experimentally obtained relationship between rotor eccentricity and the magnitude of electromagnetic force provides an important reference for the electromagnetic force that needs to be supplied for the rotor eccentricity of high-performance magnetic levitation bearings in practical applications.

The first part of this paper introduces the structure and working principle of ARMBs. In the second part, the main parameters of the ARMB are designed by establishing an equivalent magnetic circuit model, and the magnetic circuit coupling phenomenon and its influence on the magnetic field strength distribution under four magnetic pole configurations (NNSS, NSNS, NNNN, and SSSS) are analyzed by establishing a finite element model. In the third part, an experimental platform is built to detect the magnitude of electromagnetic force in the ARMB, and based on the experimental validation, not only the magnitude of electromagnetic force required to maintain the dynamic stability of the rotor under rotor eccentricity (x-axis and 45° direction) is investigated for the magnetic pole configurations of NNSS and NSNS, but the magnitude of the error between the simulated values and experimental values under the two pole configurations is also studied and compared.

## 2. Structure and Working Principle

### 2.1. Structure Composition

**Figure 1:**
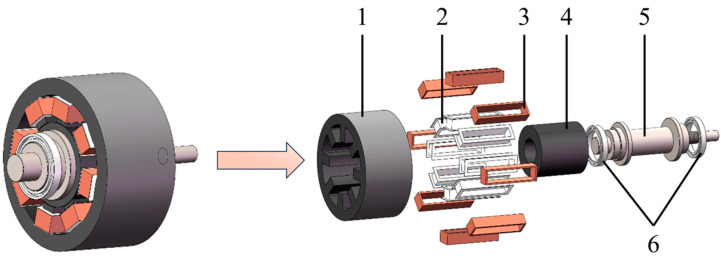
Structure of the octupole ARMB. 1—stator core, 2—fixed bracket for coil winding, 3—control coil windings, 4—rotor core, 5—shaft, 6—protective bearing.

The octupole ARMB is mainly composed of a stator and rotor. Eight magnetic poles are uniformly spaced around the stator, with the rotor located at the stator’s center. Each magnetic pole is equipped with coil windings to control the generation and change of the electromagnetic field by controlling the flow direction and intensity of the current, thereby ensuring the rotor’s stable suspension [36,37]. Figure 1 illustrates the structure of the ARMB. The coil windings are installed on eight stator poles through the coil winding fixing bracket. The rotor’s silicon steel sheet is sleeved at the outer midsection of the shaft. Protective bearings at each end of the shaft prevent the rotor’s silicon steel sheet from colliding with the stator’s silicon steel sheet when the ARMB is inactive.

### 2.2. Working Principle

The realization of ARMB technology is based on a magnetic field loop created by the electromagnet around the rotor [38]. This loop generates an electromagnetic force that dynamically attracts and stabilizes the rotor. This continuous dynamic adjustment process requires an accurate closed-loop control mechanism for continuous dynamic adjustments.

**Figure 2:**
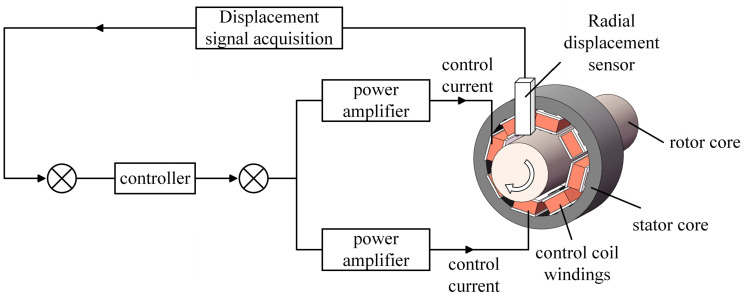
The working principle of the ARMB.

Figure 2 is the working principle of the ARMB. The radial displacement sensor first detects and captures the precise position of the rotor and converts this spatial position information into electrical signals. These signals are then received by the displacement signal acquisition. The algorithm inside the controller calculates the necessary control signals based on these input signals to adjust the control current of the stator’s coil winding in the ARMB. As the controller’s power output is not enough to directly drive the coil winding on the ARMB stator, an external power amplifier is required to enhance the current. The amplified current adjusts the coil windings, altering the magnetic field strength and subsequently adjusting the electromagnetic force applied to the rotor. This method allows for precise control of the rotor’s position, ensuring its dynamic and stable suspension.

## 3. Modelling and Design of Main Parameters

### 3.1. To Establish the Equivalent Magnetic Circuit Model of the ARMB

**Figure 3:**
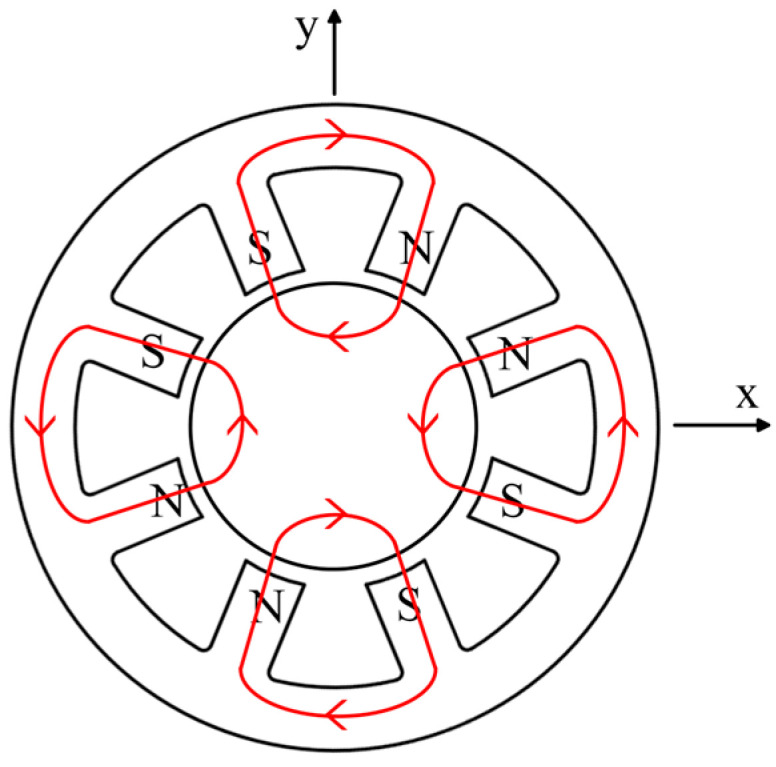
The control magnetic flux loop of the ARMB.

To improve the design efficiency of the ARMB, a mathematical model of the magnetic circuit of the ARMB is built by an equivalent magnetic circuit, before the finite element simulation. Earnshaw’s theorem states that stable levitation of the rotor in all directions cannot be achieved simultaneously by the static magnetic force between the windings and the permanent magnets alone [39]. Therefore, this instability must be realized by a control current in connection with a control system [40]. Figure 3 illustrates the ARMB control magnetic flux loop, and the red solid line represents the magnetic circuit flow direction between each pair of magnetic poles. The flow of control is produced by the control current passing through each set of stator magnetic poles. The magnetic poles of the stator can generate both N and S poles depending on the direction of the control current in the stator. The flux control loop returns from the N-pole to the S-pole, forming four closed-loop control flux paths through the N-pole stator, air gap, rotor, and S-pole stator in the eight-stage NNSS magnetic bearing. When the rotor is balanced, the control flux created by the control current in the lower air gap of each stage is the same, leading to zero combined force on the rotor. When the rotor is displaced in the +*y* direction, the control flux rises in the +*y* direction while dropping in the −*y* direction. Thus, the control flux creates a force that pushes the rotor in the +*y* direction, allowing it to revert to a new equilibrium condition.

**Figure 4:**
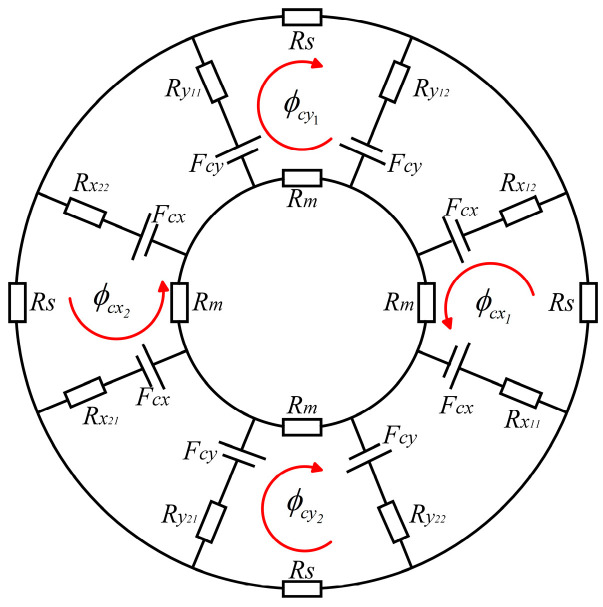
Equivalent magnetic circuit of the ARMB in the NNSS pole distribution.

Figure 4 shows the magnetic bearing control magnetic circuit loop schematized with the NNSS pole distribution. *R_m_* is the reluctance of the iron core in the rotor, *R_s_* is the reluctance at the stator yoke; $\phi_{{cx}_{1}}$, $\phi_{{cx}_{2}},\;\phi_{{cy}_{1}},\;and\;\phi_{{cy}_{2}}$ are the magnetic fluxes of the electric excitation coil winding at the magnetic bearing air gap; *F_cx_* and *F_cy_* are the magnetomotive forces generated by the radial control coil windings in the *x*- and *y*-axis, respectively; *F_x_* is the electromagnetic force in the direction of the *x*-axis degrees of freedom; *F_y_* is the electromagnetic force in the direction of the *y*-axis degrees of freedom; $R_{x}\;and\;R_{y}$ are the air gap reluctance, where the subscripts $x_{11},\;x_{12},x_{21},{\;and\;x}_{22}$ are the air gaps of the magnetic bearing in the left and right axial dimensions of the *x*-axis and the subscripts $y_{11},\;y_{12},y_{21},{\;and\;y}_{22}$ are the air gaps of the magnetic bearing in the up and down position of the *y*-axis; and $\sigma_{c}$ is the magnetic flux leakage factor in the ARMB magnetic circuit.

Supposing that the rotor displaces in the positive *x*-axis direction as *x_p_* and in the positive *y*-axis direction as *y_p_*, the magnetoresistance of individual air gaps can be formatted as follows:

(1) $$\left\{\begin{array}{c} R_{x_{11}}=R_{x_{12}}=\frac{\delta -x_{p}}{\mu_{0}A},\;R_{x_{21}}=R_{x_{22}}=\frac{\delta +x_{p}}{\mu_{0}A}\\ R_{y_{11}}=R_{y_{12}}=\frac{\delta -y_{p}}{\mu_{0}A},\;R_{y_{21}}=R_{y_{22}}=\frac{\delta +y_{p}}{\mu_{0}A}\; \end{array}\right.$$

where *δ* is the length of the one-sided air gap when the rotor maintains its central balance position, *A* is the area in the cross-section of the radial poles, and $\mu_{0}$ is the vacuum permeability.

While the rotor is in equilibrium, the magnetoresistance at the air gap can be written as follows:

(2) $$R_{x_{11}}=R_{x_{21}}=R_{y_{11}}=R_{y_{21}}=R_{\delta }=\frac{\delta }{\mu_{0}A}$$

From Figure 4, the magnetic flux and magnetic induction of the electrically excited coil at the air gap in both the X and Y dimensions are as follows:

(3) $$\left\{\begin{array}{c} \phi_{{cx}_{1}}=\frac{2F_{cx}}{\sigma_{c}(R_{x_{11}}+R_{x_{12}}+R_{m}+R_{s})},\;{\;B}_{{cx}_{1}}=\frac{\phi_{{cx}_{1}}}{A}\\ \phi_{{cx}_{2}}=\frac{2F_{cx}}{\sigma_{c}(R_{x_{21}}+R_{x_{22}}+R_{m}+R_{s})},\;{\;B}_{{cx}_{2}}=\frac{\phi_{{cx}_{2}}}{A}\\ \phi_{{cy}_{1}}=\frac{2F_{cy}}{\sigma_{c}(R_{y_{11}}+R_{y_{12}}+R_{m}+R_{s})},\;{\;B}_{{cy}_{1}}=\frac{\phi_{{cy}_{1}}}{A}\\ \phi_{{cy}_{2}}=\frac{2F_{cy}}{\sigma_{c}(R_{y_{21}}+R_{y_{22}}+R_{m}+R_{s})},\;{\;B}_{{cy}_{2}}=\frac{\phi_{{cy}_{2}}}{A} \end{array}\right.$$

According to Maxwell’s formula, the electromagnetic pull force in each degree of freedom is as follows:

(4) $$\left\{\begin{array}{c} F_{x}=\frac{\phi_{cx_{1}}^{2}-\phi_{cx_{2}}^{2}}{2\mu_{0}A}\\ F_{y}=\frac{\phi_{cy_{1}}^{2}-\phi_{cy_{2}}^{2}}{2\mu_{0}A} \end{array}\right.$$

### 3.2. Main Parameter Design

#### 3.2.1. Air Gap Bias Flux Density

The saturation flux and flux density of the iron core are denoted by $\emptyset_{s}$ and *B_s_*, respectively. To maximise the magnetic force, the control flux in Figure 4 is determined by the following equation:

(5) $$\emptyset_{cx_{1}}=\emptyset_{cy_{1}}=\emptyset_{s}/2$$

Consequently, the air gap bias flux density $B_{0}$ can be calculated by

(6) $$B_{0}=\frac{\emptyset_{s}}{2A}=\frac{B_{s}A_{teeth}}{2A}$$

where *A_teeth_* is the area of the stator teeth, *B_s_* is set as 1.4 T, and *A* is about 2 to 3 times *A_teeth_*. Therefore, *B*_0_ should be designed as 0.23~0.35 T.

#### 3.2.2. Control Current

From Figure 4, the maximum control currents can be deduced as

(7) $$I=\frac{\emptyset_{s}(R_{m}+R_{x})}{N}$$

where *N* is the turns of winding coil.

#### 3.2.3. Coil Fixing Bracket

**Figure 5:**
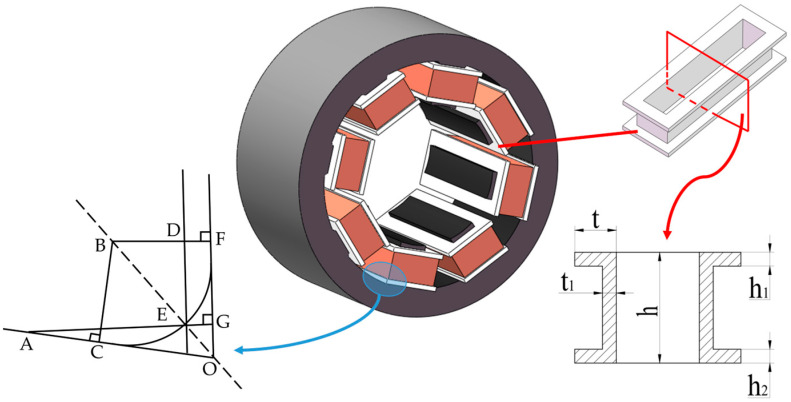
Schematic diagram of the ARMB and coil fixing bracket assembly.

Figure 5 is the assembly diagram of the coil fixing bracket in the stator of the magnetic bearing, where *h* is the height of the coil fixing bracket, *h*_1_ and *h*_2_ are the upper and lower thicknesses of the coil fixing bracket, respectively, *t* is the thickness of each side of the coil fixing bracket, and *t*_1_ is the wall thickness of each side of the coil fixing bracket. According to the structural parameters of the magnetic bearing designed above, the relationship between the structure of the coil fixed bracket and the number of turns *N* of the coil that can be accommodated can be obtained as follows:

(8) $$N\leq INT\left(\frac{t-t_{1}}{d_{c}}\right)\times INT\left(\frac{h-h_{1}-h_{2}}{d_{c}}\right)\times \lambda$$

C and F are the boundary points of the stator slot in the critical position of the coil retaining bracket assembly, B is the center of the fillet slot, and AE is the critical edge of the coil fixing bracket, which is perpendicular to the stator slot edge OF. The set coil fixed bracket and stator slot assembly gap between EG = DF = *s*, and the fillet radius BF = BC = BE = *r*. △OAG, ∠AOG=82°, from which the mathematical relationship (9) can be obtained from the critical position of *r*.

(9) $$tan82{}^{\circ}=\frac{t+s}{OF-DE}=\frac{t+s}{\frac{r}{tan41{}^{\circ}}-\sqrt{2rs-s^{2}}}$$

According to Equations (8) and (9), a coil fixing bracket is designed and the main relevant parameters are shown in Table 1.

Based on the above analysis, the main parameters of the ARMB are determined as listed in Table 2.

### 3.3. FE Model

**Figure 6:**
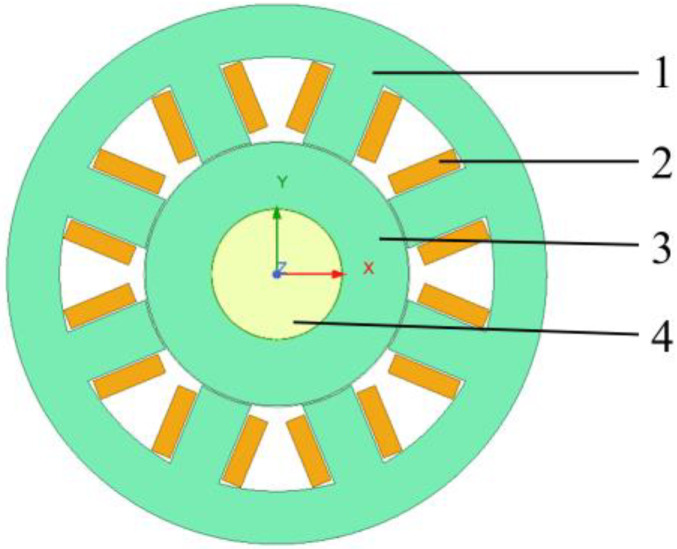
The finite element simulation model of the ARMB. 1—stator, 2—coil windings, 3—rotor, 4—shaft.

Based on the designed structural parameters of the ARMB, a 2D FEM simulation model, as depicted in Figure 6, was established. The shaft, rotor, coil winding, and stator are sequentially arranged from inside to outside. The stator and rotor of the ARMB are crafted from DW315_50, the shaft is constructed from steel, and the coil windings are composed of copper.

For comprehensive analysis of the dynamic response of magnetic levitation bearings in operation and under different rotor eccentricities, we have chosen the transient magnetic solver in the Ansys Maxwell 2022 R1 software for simulation. The transient solver was chosen for its ability to simulate the behavior of magnetic bearings under non-stationary conditions, including the transient response and long-term stability. The finite element model used in Ansys Maxwell consists of approximately 10,000 elements, which ensures a balance between computational accuracy and efficiency. In the simulation setup, a time step of 0.1 ms was set to ensure that all critical dynamic events were captured without loss of computational efficiency. In addition, we applied appropriate boundary conditions with a vector potential boundary at the outer edge of the stator with a value of 0 weber/m to simulate the actual physical environment. Then, we added the current excitation for each coil winding, and formed different magnetic poles by setting the Ref. Direction on each coil winding. Next, motion was set for the rotor as a rotation of 360 deg around the global z. Finally, the rotor displacement was set parametrically, with $move_{x}$ and $move_{y}$ being the offsets on the x- and y-axis, respectively, and this setup prepared the rotor for the later study of the relationship between the rotor eccentricity and the electromagnetic force and the magnetic flux density.

## 4. Electromagnetic Property Analysis

### 4.1. Magnetic Pole Arrangement–Magnetic Circuit Coupling Relationship

An essential component of the nonlinear spatial distribution of the electromagnetic field is the magnetic circuit coupling between the poles of the radial magnetic bearing, which has a major effect on the magnetic bearing’s overall performance [41]. The magnetic circuit connection is typically governed by the coil winding configuration of the magnetic bearing and the arrangement of its poles, both of which depend on the power supply mode.

The orientation of the magnetic poles is dictated by the direction of the current flowing into the ARMB’s stator. Based on the current flowing in different directions between the magnetic poles, four arrangements of NNSS, NSNS, NNNN, and SSSS can be formed.

**Figure 7:**
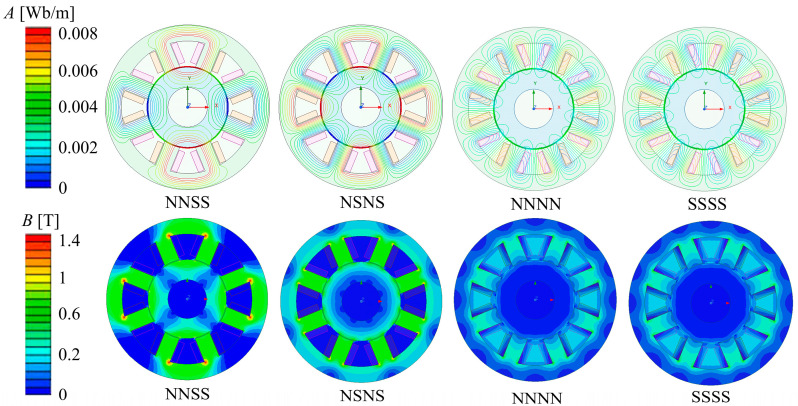
Magnetic field lines and magnetic flux density distribution of four magnetic pole forms.

Under four distinct magnetic pole arrangements, with a control current of I = 2.5 A applied to the ARMB, the magnetic induction and flux density distributions for the NNSS, NSNS, NNNN, and SSSS configurations are illustrated in Figure 7. Through the analysis, this paper explores the magnetic coupling situation between magnetic poles, the leakage phenomenon, and the magnetic field edge effect.

In the NNSS magnetic pole distribution, most magnetic lines form a closed magnetic circuit with the stator air gap and rotor between the pole pairs, with only a small number of lines passing through adjacent poles, indicating weak magnetic coupling. In the NSNS-type magnetic pole distribution, the magnetic field line forms a loop with the respective N and S magnetic poles and the neighboring S and N magnetic poles, resulting in a strong coupling between the magnetic poles.

The magnetic flux density delivery shows that in the NNSS type, the region forming the magnetic circuit has complete magnetic flux density. In contrast, the density of magnetic flux in the non-magnetic circuit region is zero, which further confirms that there is almost no coupling between adjacent magnetic pole pairs. In contrast, the NSNS-type flux not only forms a loop in the stator magnetic pole but also exists in the yoke between the magnetic poles, showing a serious magnetic coupling phenomenon.

For the NNNN and SSSS magnetic pole distribution, the magnetic field lines mostly form a loop in the air, resulting in serious magnetic flux leakage. The magnetic field strength throughout the bearing structure is quite low, which cannot effectively provide the required electromagnetic force, thereby reducing the bearing capacity.

Subsequently, the NSNS and NNSS magnetic pole distributions will be further analyzed and studied.

### 4.2. Unbalanced Disturbance of the Rotor–Magnetic Field Strength at the Air Gap Relationship

In this paper, the influence of NNSS and NSNS-type magnetic pole distributions on magnetic coupling and electromagnetic force is thoroughly analyzed through simulation. The magnetic field strength distribution when the rotor is subjected to an unbalanced offset in different directions is investigated in this paper under the conditions of a constant current. Considering the open-loop instability of the ARMB system, the rotor is susceptible to external interference during operation, resulting in a certain degree of eccentricity between the rotor and stator. It is assumed that in the previous cycle, each magnetic pole applies the constant control current to the coil winding and the rotor displacement sensor is in equilibrium. In this cycle, external load interference is applied, resulting in the offset of the rotor’s center of mass, recorded as *d*.

In this study, the *x*-axis and 45° directions are selected as typical disturbed offset directions, and the changes in magnetic flux density in these two directions are analyzed. The perturbation offset along the *x*-axis is denoted by *dx*, and that along the *y*-axis is denoted by *dy*. The magnetic flux density at the air gap is an important parameter in the design of magnetic bearings. In this paper, the variation of magnetic flux density at the air gap is investigated when the rotor is disturbed by unbalance during rotation.

**Figure 8:**
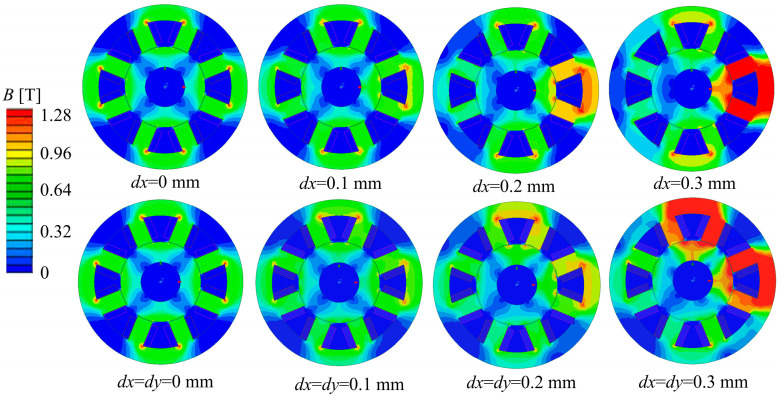
The magnetic density distribution of different disturbance offsets along the *x*-axis and 45° direction of the NNSS-type ARMB rotor.

**Figure 9:**
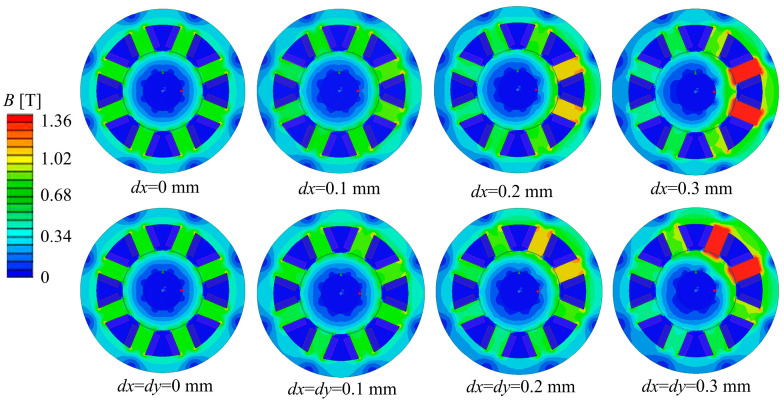
The magnetic density distribution of different disturbance offsets along the *x*-axis and 45° direction of the NSNS-type ARMB rotor.

For the NNSS and NSNS magnetic pole distributions, perturbation offsets were analyzed for *dx* and *dy* in the range of 0–0.3 mm in increments of 0.1 mm, respectively. Figure 8 and Figure 9 illustrate the magnetic density distributions for NNSS and NSNS magnetic bearings under different displacement perturbations along the *x*-axis and at a 45° angle. These figures depict the fluctuations in magnetic flux density. The results reveal that when the rotor’s displacement rises, so does the magnetic flux density in that direction. Furthermore, the distribution of magnetic flux density is symmetrical in the rotor offset direction.

**Figure 10:**
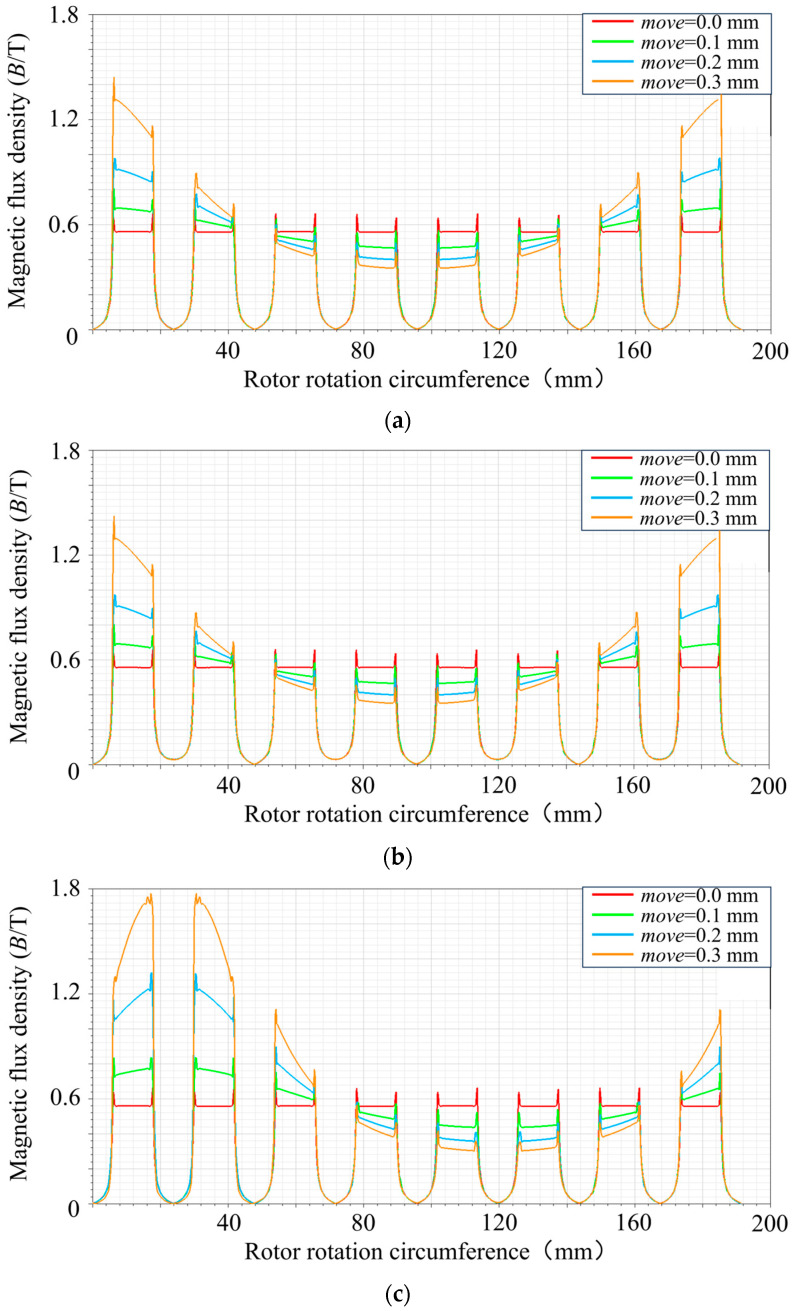

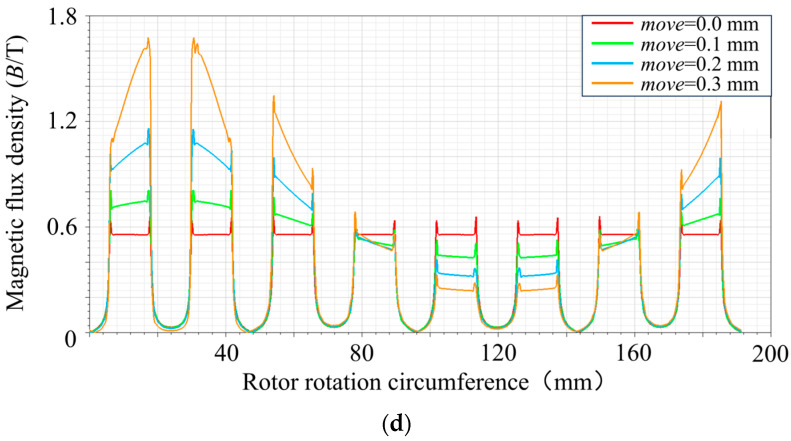
Unbalanced disturbance of the rotor–magnetic field strength at air gap relationship. (**a**) NSNS rotor offset along the x-axis in the ARMB in the form of magnetic poles. (**b**) NNSS rotor offset along the x-axis in the ARMB in the form of magnetic poles. (**c**) NSNS rotor offset along the 45° angle in the ARMB in the form of magnetic poles. (**d**) NNSS rotor offset along the 45° angle in the ARMB in the form of magnetic poles.

Figure 10 shows the magnetic field intensity curves of the air gap between the stator and rotor at different magnetic pole distributions and the magnetic field intensity curves of the rotor at different disturbance displacements. Observing Figure 10a,b, three conclusions can be reached: (1) When the rotor is off-centered by 0.1 mm in the x-axis direction, the air gap flux density of the NSNS pole arrangement is 0.46% greater than the air gap flux density of the NNSS pole arrangement. (2) When the rotor is off-centered by 0.2 mm in the x-axis direction, the air gap flux density of the NSNS pole arrangement is 0.082% greater than the air gap flux density of the NNSS pole arrangement. (3) When the rotor is off-centered by 0.3 mm in the x-axis direction, the air gap flux density of the NSNS pole arrangement is 1.13% greater than the air gap flux density of the NNSS pole arrangement. Through further observation of Figure 10c,d, the following can be observed: (1) When the rotor is off-centered by 0.1 mm in the 45° direction, the air gap flux density of the NSNS pole arrangement is 0.46% greater than the air gap flux density of the NNSS pole arrangement. (2) When the rotor is off-centered by 0.2 mm in the 45° direction, the air gap flux density of the NSNS pole arrangement is 13.79% greater than the air gap flux density of the NNSS pole arrangement. (3) When the rotor is off-centered by 0.3 mm in the 45° direction, the air gap flux density of the NSNS pole arrangement is 5.73% greater than the air gap flux density of the NNSS pole arrangement.

Comprehensive analysis shows that the magnetic flux density, within the air gap for the NSNS pole arrangement, consistently surpasses that of the NNSS pole arrangement, whether it is offset along the x-axis or 45° direction, indicating that the NSNS pole arrangement can produce greater air gap magnetic induction intensity under the constant current. When comparing changes in magnetic induction strength between the two offset directions, the offset at a 45° angle produced a significantly greater difference in magnetic induction strength between the two pole alignments (3.99%, 13.79%, and 5.73%) than the offset along the x-axis (0.46%, 0.082%, and 1.13%).

### 4.3. NNSS and NSNS Magnetic Pole Forms–Electromagnetic Force Relationship

The electromagnetic force’s magnitude is a critical parameter in designing magnetic bearing systems. The formula for calculating the electromagnetic force can be deduced from the equivalent magnetic circuit model established as follows:

(10) $$F_{e}=\frac{B^{2}}{\mu_{0}}Acos\frac{\pi }{m}$$

where *B* is the magnetic field density, $\mu_{0}$ is the permeability in vacuum, *A* is the pole area of the stator in the ARMB, and *m* is the number of stator poles in the ARMB.

**Figure 11:**
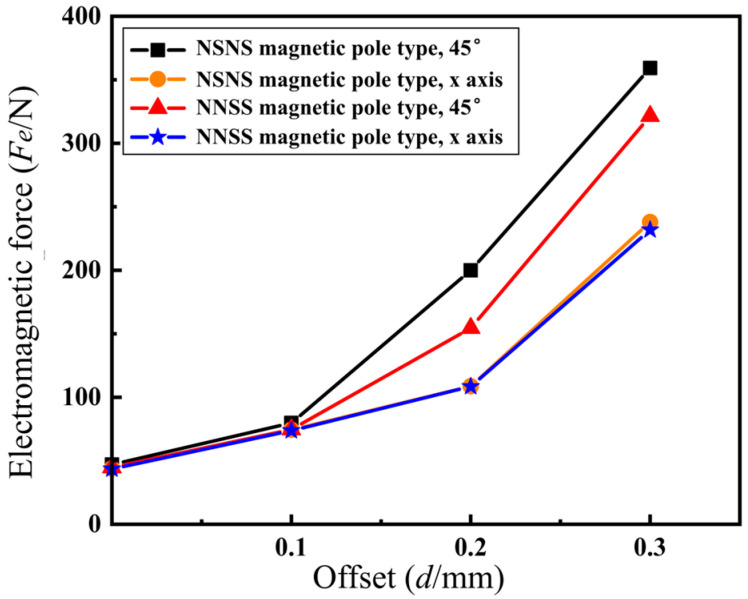
Comparison of the electromagnetic force between two different magnetic pole distribution forms.

Based on the magnitude of the flux density at the air gap corresponding to the NNSS and NSNS pole configurations at different offsets, and using (10), the rotor electromagnetic force can be calculated for different directions and offsets. Table 3 shows the computation findings. Figure 11 depicts the findings of a comparison of electromagnetic forces in the NSNS and NNSS pole configurations. By analyzing the relationship between electromagnetic force and rotor offset in these two configurations, we obtain insights into how the offset affects the electromagnetic force.

The following conclusions can be drawn from analyzing Figure 11: (1) The NSNS pole setup has a stronger electromagnetic force than the NNSS setup, whether the rotor is moved along the x-axis or at a 45° angle. (2) For both the NSNS and NNSS pole configurations, the rotor offset in the 45° direction is subjected to a greater electromagnetic force than the offset in the x-axis direction, reflecting the magnetic coupling between the coordinate axes that occurs in both pole configurations. (3) The difference in the electromagnetic force between the NNSS pole configuration in the 45° direction and the x-axis direction is smaller than that of the NSNS pole configuration, showing that the NNSS pole configuration has less difficult-to-control magnetic bearings compared to the NSNS pole configuration, and it is easier to realize the simplified control of the magnetic bearings.

## 5. Experiment and Results

### 5.1. Experimental Modeling

**Figure 12:**
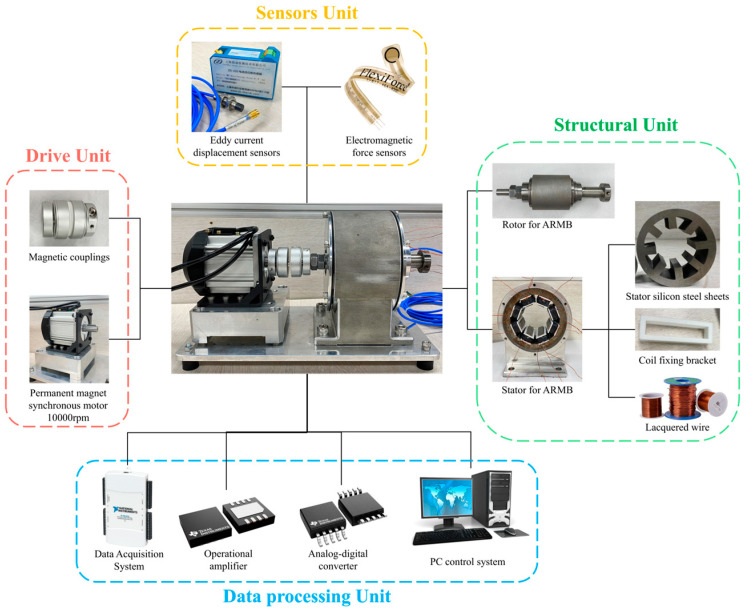
Experimental model composition of the ARMB.

The magnetic bearing electromagnetic performance experimental bench consists of a structure part, a drive part, a sensor part, and a data processing part, as shown in Figure 12. The structural part is composed of the ARMB rotor shaft and ARMB stator, which is made of 120 pieces of DW315_50 silicon steel sheets (Dongguan Hengyuan Electromechanical Technology Co., Ltd., Dongguan, China) with a thickness of 0.5 mm, and assembled in the bearing housing through interference fit. The eight coil winding seats are made of 3D printed PEEK material, and each coil winding seat is wound with 90 turns of coil winding, respectively, and the eight winding seats are installed on the eight stator teeth, respectively. The drive section consists of a permanent magnet synchronous motor with a speed of 10,000 rpm and a magnetic coupling, using which the motor can be used to drive the ARMB rotor shaft rotation without contact. The sensor part includes an eddy current displacement sensor (ZD-220, ShangHai Zhendi Detection Technology Co., Ltd., ShangHai, China), which has a linear range of 0.25~1.25 mm, a sensitivity of 16 mA/mm, and an error of 0.1%, and an electromagnetic force sensor (Tekscan FlexiForce A201, Norwood, MA, USA), which is a thin-film electromagnetic force sensor with a thickness of 0.3 mm, is suitable for use in environments with limited space, and has a measuring range of 0~1000 N, sensitivity of 0.1 N, and an accuracy of ±1–2%. The data processing part includes the data acquisition system (NI-DAQ6218, National Instruments, Austin, TX, USA), operational amplifier (INA333, Texas Instruments, Dallas, TX, USA), analogue-to-digital converter (ADS1115, Texas Instruments, Dallas, TX, USA), and a PC control system. Among these, the data acquisition system (NI-DAQ6218, National Instruments, Austin, TX, USA) has a 16-bit high resolution, which can detect subtle signal changes, and the maximum sampling rate of each channel is 250 kS/s, which is suitable for the acquisition of complex signals. The input bias current of the operational amplifier (INA333, Texas Instruments, Dallas, TX, USA) is 1 nA, which is suitable for the application of measuring small signals and can reduce error under the requirement of high precision, and it has an amplifier with very low noise characteristics, which is suitable for high-precision measurement, a high common mode rejection ratio (CMRR) of 110 dB, which can effectively suppress electromagnetic interference, enhance the accuracy of the signal, and a gain value of 1–1000. Its programmable gain and CMRR characteristics make it particularly suitable for use in high-noise environments. The ADS1115 can complete the data acquisition and provide 16-bit resolution ADC, which can be accurately converted from analogue to digital signals. The data processing and analysis software MATLAB 2016a in the PC can receive the data from the ADS1115 interface, and then set the appropriate sampling frequency and acquisition time in it to capture accurate data of the electromagnetic force changes.

**Figure 13:**
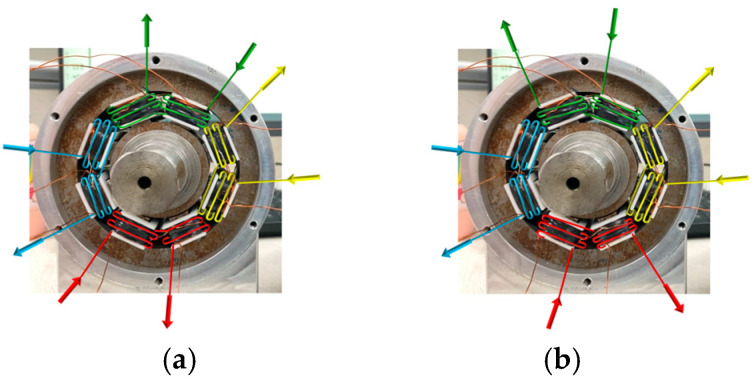
Current flow in the form of two magnetic poles. (**a**) NNSS. (**b**) NSNS.

Two directions of current flow are passed to the eight stator teeth of the magnetic levitation bearings, respectively, to form the magnetic pole forms of the NNSS type and NSNS type. The current flows of the two magnetic pole forms are shown in Figure 13, which are controlled by four independent groups of currents, with each color representing a group of currents, and the direction of the arrows representing the direction of the current flow.

### 5.2. Experimental Testing

**Figure 14:**
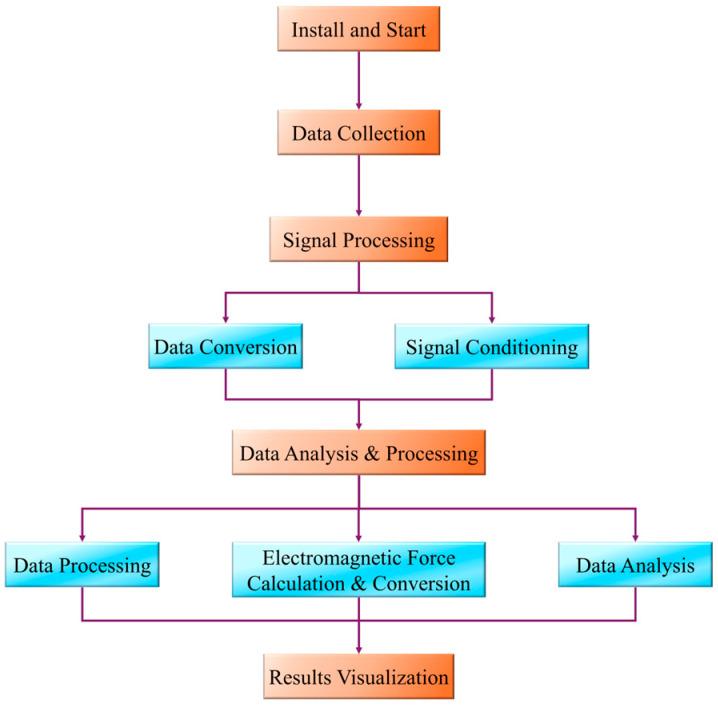
The process of experimental testing.

Figure 14 shows the flow chart of the experimental detection; the experimental detection process includes the steps of sensor installation, data acquisition, signal processing, data analysis, and result visualization. In the experiment, the detection system is constructed by installing the ZD-220 eddy current displacement sensor (ZD-220) and electromagnetic force sensor (Tekscan FlexiForce A201). Among these, the eddy current displacement sensor is installed in the form of a threaded connection on the horizontal surface of the bearing end cap, maintaining an air gap of 1 mm with the rotor, and is used to monitor the eccentricity of the rotor in real time; whereas the electromagnetic force sensor is installed on the surface of the rotor shaft in the form of surface mounting, and is used to measure the electromagnetic force applied to the rotor when it rotates to different positions. The analogue signals of these two sensors are separately acquired through the NI-DAQ 6218 data acquisition system, with the sampling frequency set to 1000 Hz and the sampling duration to no less than 10 s to ensure the integrity and fineness of the signals. After signal acquisition, the INA333 operational amplifier is used to amplify and filter the signal to improve the signal-to-noise ratio, and the analogue signal is converted into a digital signal by the ADS1115 analogue-to-digital converter. Combined with the calibration coefficients, the voltage signals from the electromagnetic force sensors are converted into actual electromagnetic force values, and the signals from the eddy current displacement sensors are converted into the eccentricity of the rotor. The data processing stage is stored and analyzed using MATLAB, where noise is removed by low-pass filtering to further optimize the signal quality and sensor-based sensitivity calculations. Finally, the relationship between rotor eccentricity and electromagnetic force in the form of the NNSS and NSNS poles is analyzed in MATLAB.

### 5.3. Experimental Results and Analysis

Through the above detection method, the size of the electromagnetic force generated when the rotor is offset in the x-axis and 45° direction by different amounts under the two magnetic pole forms of five groups of the magnetic levitation bearings NNSS and NSNS are detected, and the experimental results are shown in Table 4. Analyzing the average value of the detection results of each experimental group and the value of the electromagnetic force simulation results obtained in Chapter 4, it can be concluded that the inaccuracy between the experimental and simulation results is within 10%, which is in line with the requirements of the reasonable range of experimental inaccuracy, indicating that the experimental detection scheme can effectively verify the correctness of the simulation model. Moreover, the experimental and simulation errors of the NNSS magnetic pole form are smaller than those of the NSNS magnetic pole form, indicating that the experimental results of the NNSS magnetic pole form are closer to the simulation values, which suggests that this magnetic pole form is easier to model accurately and can provide more reliable simulation data for the actual work of the magnetic levitation bearing.

**Figure 15:**
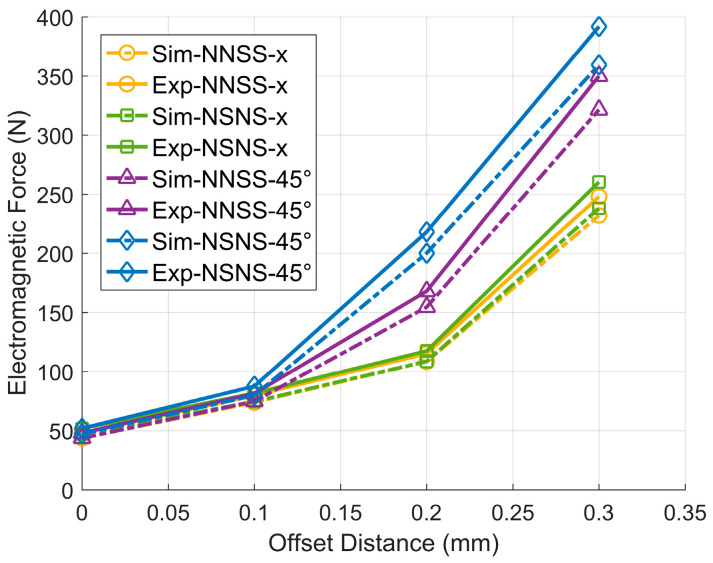
Plot of the data comparison between the experiment and simulation.

Figure 15 shows the comparison curve of the experimental results with the simulation results, in which Sim indicates the simulation results and Exp indicates the experimental results. Through the analysis, the following can be concluded: (1) The data change trends of the simulation results and experimental results are the same, and the curve of the simulation results is very close to the curve of the experimental results, which verifies the accuracy of the experimental method and the reliability of the detection of the electromagnetic force sensor. (2) The spacing between the experimental and simulation curves is smaller in the form of the NNSS magnetic poles than in the form of the NSNS magnetic poles, which indicates that the symmetry of the magnetic circuit in the form of NNSS is better, the nonlinearities and the edge effects are smaller, and it is easier to model accurately. In the NSNS form, the magnetic field distribution is complicated due to the magnetic poles with the same adjacent polarity, and it is easily affected by the saturation of the magnetic circuit and the edge effects, which leads to the large simulation error. It shows that the NNSS pole form is easier to model accurately and can provide more reliable simulation data for the actual work of maglev bearings. (3) The measured electromagnetic force is larger than the simulation result, which may be due to the geometrical error of the rotor and stator in the experimental setup and the difference of the coil arrangement leading to the increase of the electromagnetic force, or it may also be that the simulation does not consider the saturation effect of the silicon steel sheet under the high current or the high magnetic field intensity completely.

A comparison of the experimental and simulation data reveals that while finite element simulations provide valuable insights into the properties of electromagnetic forces under different pole configurations, they are subject to limitations inherent to computational methodology, such as material property assumptions, mesh resolution, and the neglect of certain dynamic effects. In finite element simulations, the accuracy of meshing has a significant impact on the accuracy of the results. Although a mesh division of approximately 10,000 cells was used in this paper to balance computational efficiency and accuracy, finer meshes may further improve the accuracy of the results but also significantly increase the computational resource requirements. The properties of magnetic materials used in simulations are usually based on idealized conditions, whereas real materials may exhibit different properties under dynamic and complex operating conditions, such as hysteresis effects and the influence of temperature on magnetic parameters. Certain dynamic coupling effects, such as gyroscopic and aerodynamic effects of the rotor at high rotational speeds, are not fully considered in the simulation models, which may significantly affect the distribution and stability of the electromagnetic force in real operating conditions.

In order to further improve the reliability of the model, the simulation model can be corrected and optimized in future work by combining the experimental data under dynamic working conditions, introducing the nonlinear properties of magnetic materials and temperature effects into the simulation to be closer to the actual conditions, and exploring multi-physics field coupling simulation (e.g., magneto-thermal coupling and mechanical coupling) in order to study the performance under complex working conditions.

## 6. Conclusions

In this paper, an ARMB is designed using the equivalent magnetic circuit method, the electromagnetic performance of four magnetic pole configurations (NNSS, NSNS, NNNN, and SSSS) is analyzed by a finite element simulation system, and the differences in the electromagnetic force applied to the rotor when the rotor is eccentrically oriented in the x-axis direction and in the 45° direction under the two pole configurations, NNSS and NSNS, are highlighted by experiments. The simulation results show that the NNSS configuration significantly reduces the magnetic circuit coupling phenomenon and improves the efficiency of electromagnetic force utilization compared with other configurations. The experimental results show that the deviation between the experimental measured and simulated values of the electromagnetic force in the NNSS configuration is less than 10%, which verifies the reliability and practicality of the design. The reason for the errors may be due to the increased electromagnetic force caused by the geometrical errors of the rotor and stator in the experimental setup and the differences in the coil arrangement, or the saturation effect of the silicon steel sheet under high currents or high magnetic field strengths not being fully considered in the simulation. Future research will aim to incorporate more comprehensive physical models and validate them under a wider range of experimental conditions to further improve the reliability of the simulations. The relationship between rotor eccentricity and the magnitude of electromagnetic force obtained in the experiments provides an important reference for the electromagnetic force to be supplied by the high-performance magnetic levitation bearings in practical applications where rotor eccentricity occurs. This validation result further demonstrates that the NNSS configuration can simplify control strategy, reduce design complexity, and enhance operational efficiency.

## Figures and Tables

**Table 1:** Coil fixed bracket parameters.

Parameter	Value
Height of coil fixing bracket, *h*	14 mm
Top thickness of coil fixing bracket, *h*_1_	0.3 mm
Bottom thickness of coil fixed support, *h*_2_	0.4 mm
Thickness of each side of coil fixing bracket, *t*	3.3 mm
Wall thickness of each side of coil fixing bracket, *t*_1_	0.3 mm

**Table 2:** Structure parameters of the ARMB.

Parameter	Value
Inner diameter of the stator core, *d*	61 mm
Outer diameter of the stator core, *D*	123 mm
Width of the magnetic pole, *b*	12 mm
Width of the yoke, *c*	12 mm
Diameter of the rotor, *D*_0_	60 mm
Radial air gap length, *δ*_0_	0.5 mm
Bias flux density, *B*_0_	0.3 T
Control current of the coil, *I*	2.5 A
Coil turns of each magnet, *N*	90
Occupy ratio, *λ*	0.7

**Table 3:** Electromagnetic force at different offsets.

**x-Axis Direction Offset (** ***d_x_*** **)/mm**		**0**	**0.1**	**0.2**	**0.3**
$F_{e}/N$	NSNS	47.082	74.431	108.637	237.957
	NNSS	43.541	73.749	108.459	231.827
**45° Direction Offset (** ***d_x_*** **= *d_y_*** **)/mm**		**0**	**0.1**	**0.2**	**0.3**
$F_{e}/N$	NSNS	47.082	79.725	199.980	359.214
	NNSS	43.541	74.652	154.459	321.351

**Table 4:** Experimental data on the electromagnetic force applied at different offsets.

Form	Offset (mm)	Direction	Exp1 (N)	Exp2 (N)	Exp3 (N)	Exp4 (N)	Exp5 (N)	Average (N)	Inaccuracy
NNSS	0		47.461	47.146	48.876	49.013	46.482	47.796	9.77%
	0.1	x-axis	81.125	80.519	78.034	79.481	79.346	79.701	8.07%
		45°	81.565	81.022	80.571	81.543	82.006	81.341	8.96%
	0.2	x-axis	118.618	115.951	102.115	120.008	119.171	115.173	6.19%
		45°	169.005	167.375	169.852	165.844	170.489	168.513	8.71%
	0.3	x-axis	239.771	249.249	250.186	248.005	252.713	247.984	6.97%
		45°	348.265	352.146	349.855	352.564	350.112	350.589	8.85%
NSNS	0		51.781	52.016	50.842	51.143	53.004	51.757	9.93%
	0.1	x-axis	82.010	81.941	81.636	80.841	81.007	81.487	9.47%
		45°	87.477	86.748	87.512	87.228	88.014	87.396	9.62%
	0.2	x-axis	118.871	115.657	113.180	119.204	120.006	117.384	8.05%
		45°	218.633	218.418	216.984	217.511	218.010	217.910	8.97%
	0.3	x-axis	261.875	258.831	261.146	260.664	259.042	260.312	9.39%
		45°	390.517	389.772	390.159	392.114	390.106	390.534	9.08%

## Data Availability

Data are contained within the article.
